# Stunting and severe stunting among infants in India: the role of delayed introduction of complementary foods and community and household factors

**DOI:** 10.1080/16549716.2019.1638020

**Published:** 2019-07-23

**Authors:** Mansi Vijaybhai Dhami, Felix Akpojene Ogbo, Uchechukwu L. Osuagwu, Zino Ugboma, Kingsley E. Agho

**Affiliations:** a Translational Health Research Institute (THRI), School of Medicine, Western Sydney University, Campbelltown Campus, Penrith, Australia; b General Practice Unit, Prescot Specialist Medical Centre, Makurdi, Nigeria; c School of Medicine | Diabetes Obesity and Metabolism Translational Research Unit (DOMTRU), Macarthur Clinical School, Campbelltown, Australia; d Faculty of Law, Baze University, Abuja, Nigeria; e School of Science and Health, Western Sydney University, Campbelltown Campus, Penrith, Australia

**Keywords:** Stunting, delayed introduction of complementary feeding, India, infants, nutrition

## Abstract

**Background**: Delayed introduction of solid, semi-solid or soft foods (complementary feeding) and associated factors are related to stunting and severe stunting among children in many low- and middle-income countries. In India, however, there is limited evidence on the relationship between delayed complementary feeding and associated factors with stunting and severe stunting to advocate for policy interventions.

**Objectives**: The present study investigated the relationship between delayed complementary feeding and associated factors with stunting and severe stunting among infants aged 6–8 months in India.

**Methods**: Survey data on 13,548 infants aged 6–8 months were obtained from the 2015–16 National Family Health Survey in India. Logistic regression (Generalized Linear Latent and Mixed Models [GLLAMM] with a logit link and binomial family) models that adjusted for clustering and sampling weights were used to investigate the relationship between delayed complementary feeding and associated factors (community, household, maternal, child and health service factors) with stunting and severe stunting among infants aged 6–8 months in India.

**Results**: The prevalence of stunting and severe stunting was 22.0% (95% CI: 21.0–23.7%) and 10.0% (95% CI: 9.0–11.0%) among infants aged 6–8 months who received no complementary foods, respectively. Delayed introduction of solid, semi-solid or soft foods was associated with stunting (adjusted Odd ratios [aOR] = 1.24, 95% CI: 1.09–1.41) and severe stunting (aOR = 1.21, 95% CI: 1.01–1.45) among infants aged 6–8 months. High maternal education (secondary or higher education) and household wealth (middle, richer and richest) were protective against stunting and severe stunting.

**Conclusion**: Delayed introduction of complementary foods and associated factors were related to stunting and severe stunting among infants aged 6–8 months in India. Reducing the proportion of infants who are stunted in India would require comprehensive national nutrition policy actions that target the sub-population of mothers with no schooling and limited resources.

## Background

The introduction of solid, semi-solid or soft foods (complementary feeding) is essential for the infant’s optimal growth and development, particularly after the first six months of life as breast milk alone is no longer sufficient for maintaining the infant’s nutritional and developmental needs [1]. In India, a recent report has indicated that only about 55% of mothers introduced complementary foods to their infants as recommended by the World Health Organization (WHO, that is, the introduction of complementary food to infants aged 6–8 months) [2]. Inappropriate introduction of complementary foods is associated with adverse child health outcomes and can have subsequent effects across the life span [2–4]. Past studies from India have suggested that lower maternal education, poor socioeconomic status and limited health service access were associated with the inappropriate introduction of complementary foods [2,5,6].

Stunting, defined as height-for-age less than minus two standard deviations of the WHO Child Growth Standards median [7], is prevalent in many low- and middle-income countries (LMICs), including India. In those countries, under-five mortality is also prevalent, and malnutrition (i.e. stunting, wasting and underweight) may be attributable to more than half of those deaths, highlighting the substantial negative impact of inappropriate nutrition among children in those contexts [8]. Although recent evidence indicates that the prevalence of stunting has reduced from 198 million in 2000 to 151 million in 2017, approximately one in four children younger than 5 years were stunted globally in 2017 [8]. In South Asia, approximately 38% of children younger than 5 years were stunted in 2017 [9], in which India was the largest contributor (48%), followed by Pakistan (43%), Bangladesh (41%), Nepal (41%) and Sri Lanka (19%) [7].

In India, evidence from discrete subnational studies suggested that stunting was associated with an array of factors. These factors included delayed initiation of breastfeeding [10], colostrum deprivation [11], delayed introduction of complementary foods [11], female gender [12], maternal underweight [13], a lack or fewer than recommended antenatal visit [14], higher birth order [12], lower socioeconomic status [13,15], a lack or lower maternal education [13,15] and a lack of potable water and poor hygiene [16], as well as household food competition [13,17]. Additionally, a national level study conducted in India also indicated that age-appropriate infant and young child feeding practices were associated with child undernutrition [18]. However, while studies from India have reported a high prevalence of delayed introduction of complementary foods [19], as well as a high proportion of stunting among children younger than five years [20,21], there has been no nationally representative studies that investigated the association between delayed introduction of solid, semi-solid or soft foods and associated factors with stunting in India, nor has there been a documented prevalence of stunting by severity. Nationally representative studies in this context are needed as they provide complete information, as well as highlight the characteristics of childhood stunting to match the country’s policy directives and interest [22]. Therefore, evidence on the association between the delayed introduction of complementary foods and stunting would be essential to nutrition experts and public health planners in India to help refine current infant and young child (IYCF) policies and interventions to high-priority groups in order to reduce the proportion of stunted children in India.

The present study aimed to investigate the relationship between the delayed introduction of solid, semi-solid or soft foods and stunting and severe stunting among infants aged 6–8 months in India. The study also reports on the association between other potential factors (community, socio-demographic and health service factors) and stunting by severity to provide a broader picture of factors influencing stunting to inform targeted interventions in one of the world’s largest population.

## Methods

### Data sources

The study used data from the 2015–16 National Family Health Survey (NFHS–4) conducted by International Institute for Population Sciences, Mumbai, India. The Ministry of Health and Family Welfare (MoHFW), Government of India supervised the survey, and technical assistance was provided by the United States Inner City Fund (ICF) International, Maryland. Information on socio-demographic and household status, as well as IYCF data, were collected from a sample of women aged between 15 and 49 years. The response rates were high during data collection, ranging 94.0–99.6% in various states and territories in India, including Andhra Pradesh (94.0%), West Bengal (94.0%) and Bihar (99.6%) [15].

In the survey, approximately 572,000 Indian households were selected from 249,454,252 population using the 2011 census frame that covered each of the 29 states and 7 union territories of India. Each district had a sample based on its size for producing a reliable indicator. Rural areas were administered with a two-stage sampling design. In these areas, the first stage involved the villages (selected based on the probability proportional to the size of the sample) being designated as Primary Sampling Units (PSUs) and the second stage involved a random selection of 22 households. A similar two-stage sampling approach was used in urban areas. The first stage involved the selection of Census Enumeration Blocks (CEBs) and the second stage involved a random selection of 22 CEBs [23]. Additional information on the sampling procedure is provided in the national report of the NFHS-4 [24]. In this study, the analyses were restricted to the youngest living children aged 6–8 months, living with the respondent (women aged 15–49 years) to reduce the potential impact of recall bias, consistent with previously published studies [3,25], yielding a total weighted sample of 13,548 infants.

### Outcome variables

The primary outcome variables were stunting and severe stunting, consistent with previously published studies [26–28]. Evidence from LMICs suggests that there is an increased risk of child morbidity and mortality with a varied degree of stunting [29–31] with implication for policy interventions [32]. The outcome variables were expressed as a dichotomous variable. Stunting- was categorised as ‘0’ [not stunted (height-for-age (HAZ) greater than −2 standard deviations (SD)] of the WHO Child Growth Standards median and ‘1’ [stunted (HAZ less than −2SD)]. Severe stunting was expressed as ‘0’ [not severely stunted (HAZ greater than −3SD)] and ‘1’ [severely stunted (HAZ less than −3SD)] [33].

### Exposure variables

The main exposure variable was the delayed introduction of solid, semi-solid or soft foods among infants aged 6–8 months. This was defined in accordance with the WHO guidelines for assessing IYCF practices in the population [1]. In this study, infants aged 6–8 months who appropriately received solid, semi-solid or soft foods were coded as ‘1’, and those who did not receive similar foods were coded as ‘0’ (delayed introduction of complementary foods).

Based on previously published studies [14,28,34–36] and data availability, associated factors examined in the study included community, household, maternal, child and health service factors. Community-level characteristics included geographical regions and area of residence was categorised as urban or rural. For the family or household level attributes, individuals’ marital status and household wealth index [15] were considered. The household wealth index was classified into five quintiles (categories) by the IIPS and ICF International, which included poorest, poorer, middle, rich and richest. Maternal characteristics included variables such as age, education and literacy level [37], body mass index (BMI) [37], employment status, type of caste or tribe and religion. Individual factors of the child included sex [15], birth order [14], the perceived size of the baby at birth [36], and the preceding birth interval. Furthermore, the number of visits to ANCs (none, 1 to 3 visits or ≥ 4 visits) [14], place of delivery (health facility or home), type of delivery assistance such as presence of a health care professional or a traditional birth attendant and mode of delivery (caesarean or non-caesarean) were also considered.

### Statistical analysis

In this study, we used STATA/MP version 14 (Stata Corp, College Station, TX, USA) to conduct all statistical analyses. The ‘svy’ command was employed to allow for adjustments for the cluster-sampling design and weight to calculate frequencies and prevalence. We first conducted frequency tabulations for all exposure variables, and this was followed by univariate analyses that independently examined the relationship between stunting and severe stunting by the delayed introduction of complementary foods. Multivariate analyses were used to examine the relationship between the delayed introduction of complementary foods and stunting and severe stunting using logistic regression Generalized Linear Latent and Mixed Models (GLLAMM) with a logit link and binomial family. As part of the multivariate analyses, a staged modelling technique was employed.

As a process of multivariate modelling technique, all community and household level factors were first entered into the baseline multivariate model with the elimination process to remove statistically non-significant variables (Model 1). In the next stage, maternal factors were examined with model 1, variables that were significantly associated with the outcomes were retained as (Model 2). Next, child factors were assessed with model 2 and associated factors were retained as (Model 3). In the fourth modelling stage, a similar procedure was used for health services factors and those variables with p-values <0.05 were retained as (Model 4). In the final model (Model 5), the exposure variable (delayed introduction of complementary foods) was examined with those variables significant in Model 4 and elimination process were used to retain those variables with p-values <0.05. In the final model,

we tested the interaction between complementary feeding and mother education and household wealth index and reported any co-linearity. The odds ratios with 95% confidence intervals were calculated to assess the association between the independent variables with the study outcomes.

## Results

### Characteristics of the study population

In the study population, many mothers (58.5%) had secondary and higher education, 13.3% had primary education, and 28.1% had no education. The majority (89.2%) of the mothers were unemployed and most women resided in rural areas compared to the urban areas (72.8% versus 27.1%) [Table 1].

**Table 1.** Characteristics of the study population (N = 13,548).

Characteristic	n	%
***Community level factors***		
**Geographical region**		
North	1746	12.8
South	2288	16.8
East	3536	26.1
West	1684	12.4
Central	3910	28.8
North East	384	2.8
**Residence**		
Urban	3683	27.1
Rural	9863	72.8
***Household characteristics***		
**Household wealth index**		
Poorest	3366	24.8
Poorer	3012	22.2
Middle	2712	20.0
Richer	2439	18.0
Richest	2017	14.8
**Source of drinking water**		
Improved	11411	84.23
Not improved	2136	15.77
***Maternal Characteristics***		
**Mother’s age**		
15–19 years	923	6.8
20–34 years	11958	88.2
35–49 years	666	4.9
**Mother’s education**		
No education	3808	28.1
Primary	1803	13.3
Secondary and higher	7936	58.5
**Employment**		
Did not work	2116	15.6
Worked	254	1.8
**Mother’s religion**		
Hindu	10726	79.1
Muslim	2148	15.8
Christianity and others	673	4.9
**Type of Caste or tribe**		
Scheduled Caste	3101	22.8
Scheduled tribe	1376	10.1
Other backward class	5921	43.7
Others^$^	3148	23.2
***Child Characteristics***		
**Sex of baby**		
Male	7312	53.9
Female	6235	46.0
Perceived Size of baby		
Small	1728	12.7
Average	8960	66.1
Large	2695	19.8
***Health Service characteristics***		
**Antenatal clinic visits**		
None	2228	16.4
1–3	4576	33.7
4+	6588	48.6
**Mode of delivery**		
Non-Caesarean	10961	80.9
Caesarean	2586	19.1
***Study outcomes***		
**Stunting (<- 2 SD)**		
Not stunted	9143	67.4
Stunted	2325	17.1
**Severe stunting (<- 3 SD)**		
Not severely stunted	10430	76.9
Severely stunted	1038	7.6

n = weighted counts; ^$^ = no caste or tribe/don’t know)

### Relationship between the delayed introduction of complementary foods and stunting and severe stunting

**Figure 1.**
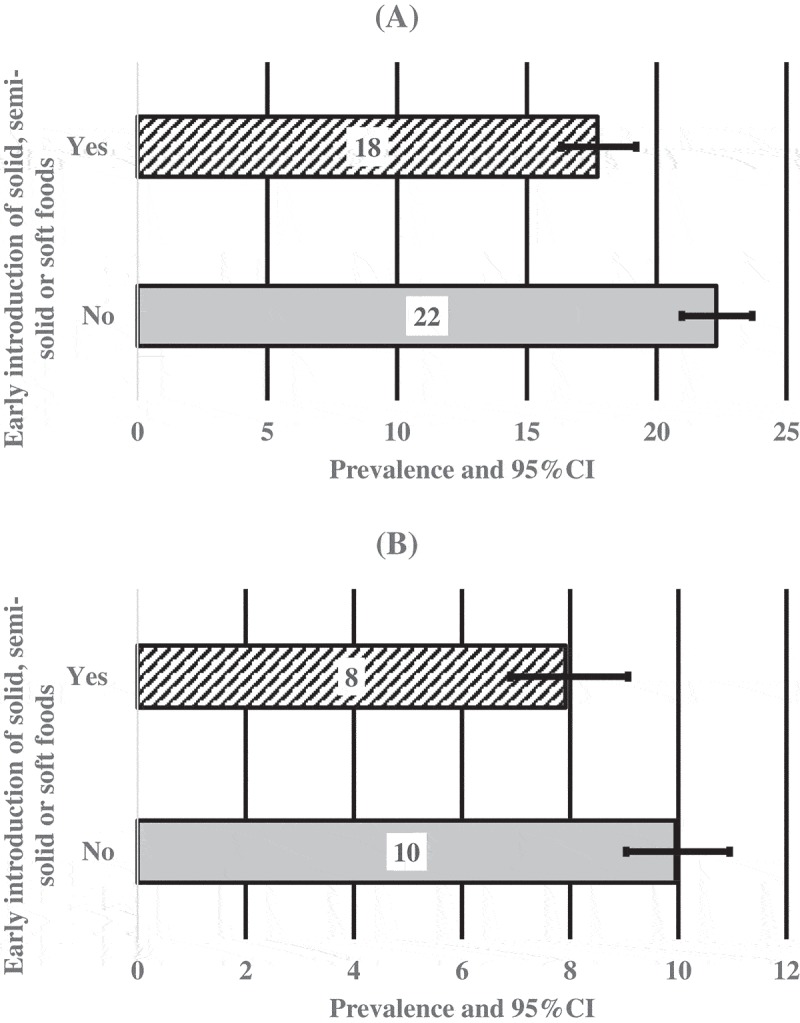
Prevalence and 95% Confidence Intervals (CI) of stunting (A) and severe stunting (B) by the introduction of solid, semi-solid or soft foods among infants aged 6–8 months in India.

The prevalence of stunting was 22.0% (95% confidence interval [95% CI]: 21.0–23.7%) and that for severe stunting was 10.0% (95% CI: 9.0–11.0%) among infants aged 6–8 months who received no complementary foods in India [Figure 1]. The study indicated that delayed introduction of solid, semi-solid or soft foods was associated with both stunting (aOR = 1.24, 95% CI: 1.09–1.41) and severe stunting (aOR = 1.21, 95% CI: 1.01–1.45) [Table 2].

**Table 2.** Factors associated with stunting among infants aged 6–8 months in India, NFHS-4.

Variables	OR [95% CI]	*P-value*	aOR^¥^ [95% CI]	*P-value*
Introduction of solid, semi-solid or soft foods				
Early introduction of foods	1.00		1.00	
Delayed introduction of foods	1.33 (1.18, 1.51)	<0.001	1.24(1.09, 1.41)	0.001
Household Wealth Index				
Poorest	1.00		1.00	
Poorer	0.76(0.65, 0.89)	0.001	0.83(0.70, 0.98) *	0.029
Middle	0.58(0.49, 0.70)	<0.001	0.66(0.54, 0.80) *	<0.001
Richer	0.47(0.38, 0.58)	<0.001	0.54(0.43, 0.68) *	<0.001
Richest	0.38(0.30, 0.48)	<0.001	0.46(0.36, 0.61) *	<0.001
Mother’s education				
No education	1.00		1.00	
Primary	0.80(0.66,0.98)	0.031	0.90(0.74, 1.09)	0.295
Secondary and higher	0.58(0.50, 0.66)	<0.001	0.81(0.69, 0.94) *	0.008
Type of Caste or tribe				
Scheduled Caste	1.00		1.00	
Scheduled tribe	0.88(0.72, 1.07)	0.217	0.79(0.64, 0.96) *	0.024
Other backward class (OBC)	0.85(0.73, 0.99)	0.04	0.90(0.77, 1.05)	0.196
Others^$^	0.65(0.53, 0.79)	<0.001	0.79(0.64, 0.97) *	0.031
Sex of baby				
Male	1.00		1.00	
Female	0.78(0.69, 0.88)	<0.001	0.75(0.66, 0.84) *	<0.001
Birth order				
Firstborn	1.00		1.00	
2^nd^–4^th^	1.13(0.992, 1.307)	0.063	1.02(0.89, 1.18)	0.727
5 or more	1.68(1.35, 2.08)	<0.001	1.17(0.92, 1.48)	0.186
Perceived Size of baby				
Small	1.00		1.00	
Average	0.60(0.50, 0.72)	<0.001	0.62(0.51, 0.74) *	<0.001
Large	0.48(0.38, 0.61)	<0.001	0.50(0.40, 0.64) *	<0.001

*Statistically significant (95% confidence intervals and P < 0.05) study variables from multivariate models are shown. Multivariate models adjusted for child, maternal, household, health service and community factors. OR: odds ratios; aOR: adjusted odds ratios; 95%CI: 95% confidence interval; ^$^ = no caste or tribe/don’t know)

### Other factors associated with stunting and severe stunting

The household wealth index was a significant predictor of stunting among infants such that infants who were born to the richest, richer, middle and poorer households were less likely to be stunted compared to those born in poorest households (aOR = 0.46, 95% CI: 0.36–0.61 for richest, aOR = 0.54, 95% CI: 0.43–0.68 for richer, aOR = 0.66, 95% CI: 0.54–0.80 for middle and aOR = 0.83, 95% CI: 0.70–0.98 for poorer households) [Table 2]. Mothers who had secondary or higher education were less likely to have babies who were stunted compared to those with no schooling (aOR = 0.81, 95% CI: 0.69–0.94). The mothers’ type of caste was associated with stunting such that infants from scheduled tribe or others (no caste or tribe) were less likely to be stunted compared to those in scheduled caste (aOR = 0.79, 95% CI: 0.64–0.96 for scheduled tribe and aOR = 0.79, 95% CI: 0.64–0.96 for others). Large or average sized babies were less likely to be stunted compared to infants who were perceived to be small (aOR = 0.50, 95% CI: 0.40–0.64 for large-sized babies and aOR = 0.62, 95% CI: 0.51–0.74 for average sized babies, respectively) [Table 2].

Multivariate analyses assessing factors associated with severe stunting indicated that female infants were less likely to be severely stunted compared to male infants (aOR = 0.67, 95% CI: 0.56–0.80) [Table 3]. Infants whose mothers perceived to be large and average-sized infants were less likely to be severely stunted compared to small sized infants (aOR = 0.63, 95% CI: 0.46–0.85 and aOR = 0.64, 95% CI: 0.51–0.82 for large and average-sized infants, respectively). Lower household wealth index was related to severe stunting compared to higher household wealth index (aOR = 0.70, 95% CI: 0.55–0.90 for middle, aOR = 0.68, 95% CI: 0.51–0.92 for the richer, aOR = 0.52, 95% CI: 0.37–0.74 for the richest) [Table 3]. Infants who were delivered by caesarian section were less likely to be severely stunted compared to those who were delivered vaginally (aOR = 0.69, 95% CI: 0.50–0.95).

**Table 3.** Factors associated with severe stunting among infants aged 6–8 months in India, NFHS-4.

Variables	OR [95% CI]	*P-value*	aOR [95% CI]	*P-value*
Introduction of solid, semi-solid or soft foods				
Early introduction of foods	1.00		1.00	
Delayed introduction of foods	1.29 (1.07, 1.54)	0.007	1.21(1.01, 1.45)	0.039
Household Wealth Index				
Poorest	1.00		1.00	
Poorer	0.78(0.62, 0.99)	0.047	0.81(0.64, 1.03)	0.093
Middle	0.67(0.52, 0.85)	0.001	0.70(0.55, 0.90) *	0.006
Richer	0.63(0.47, 0.84)	0.002	0.68(0.51, 0.92) *	0.012
Richest	0.46(0.32, 0.67)	<0.001	0.52(0.37,0.74) *	<0.001
Sex of baby				
Male	1.00		1.00	
Female	0.69(0.57, 0.82)	<0.001	0.67(0.56, 0.80) *	<0.001
Perceived size of baby				
Small	1.00		1.00	
Average	0.63(0.50, 0.80)	<0.001	0.64(0.51, 0.82) *	<0.001
Large	0.60(0.44, 0.81)	0.001	0.63(0.46, 0.85) *	0.003
Mode of delivery				
Non-caesarian	1.00		1.00	
Caesarian	0.59(0.43, 0.82)	0.001	0.69(0.50, 0.95) *	0.023

*Statistically significant (95% confidence intervals and P < 0.05) study variables from multivariate models are shown. Multivariate models adjusted for child, maternal, household, health service and community factors. OR: odds ratios; aOR: adjusted odds ratios; 95%CI: 95% confidence interval

Overall, the interaction between complementary feeding and household wealth index was significant for both stunting and severe stunting. In contrast, there were no significant associations between the delayed introduction of complementary foods and mother education with stunting and severe stunting.

## Discussion

The present study suggested that 22.0% and 10.0% of infants aged 6–8 months who had delayed complementary foods were stunted and severely stunted, respectively. Delayed introduction of solid, semi-solid or soft foods was associated with stunting and severe stunting among infants aged 6–8 months in India. Factors associated with a reduced risk of stunting included higher maternal education, belonging to wealthier households, and perceived size (average or large) of the baby at birth. Female sex, higher household wealth, perceived size (large and average) of the baby at birth and caesarean births were associated with a reduced risk of severe stunting in India.

Globally, an estimated 3 million deaths among children younger than five years were due to malnutrition in 2018 [8], reflecting the burden of stunting, wasting and underweight among young children. In India, a recent study indicated that about 0.9 million under-5 deaths occurred in 2016, with malnutrition (including stunting) playing an important role [38]. In India, previous small-scale studies have estimated the prevalence of stunting to be as high as 48% [39] and 51% [10], possibly reflecting variations and/or limitations in sample size or the ‘true’ burden of stunting in those populations. India is a country with significant variations in socio-economic status, culture and geographical boundaries, with factors such as maternal education and household wealth playing an influential role in infant nutritional status, including stunting.

Our study suggested that delayed introduction of solid, semi-solid or soft foods was associated with stunting and severe stunting in infants aged 6–8 months. This finding is consistent with studies from regional India [10,39], which indicated that children whose mothers delayed the introduction of complementary foods were more likely to be stunted. In contrast, a study conducted in Bangladesh found that the age of introducing complementary feeding was not associated with the linear growth of the infant [40]. However, the study concluded that optimal complementary feeding practice might play an important role in reducing the prevalence of stunting in the second year of a child’s life [40]. Evidence suggests that inadequate nutrition in the form of delayed complementary feeding compromises the quality and/or quantity of infant’s dietary requirements which in turn results in stunting [41]. Specifically, inappropriate complementary feeding (from poor hygiene and *mycotoxicity* due to incorrect food handling and storage) can result in subclinical infections which alter the gut microbial flora and may result in a change in the barrier mechanism of the gut, with subsequent negative impacts on optimal nutrient absorption [41]. This potentially reflects one of the rationales for why the WHO recommends that the introduction of complementary foods to infants should be timely, adequate, safe and appropriate [42].

The present study found that higher maternal education was associated with a reduced risk of stunting in infants aged 6–8 months. This finding is consistent with previous studies from Kenya [43], and China [35], which demonstrated that higher maternal education was associated with a reduced risk of stunting in infants. Additionally, a study from Pakistan also indicated that stunting in children younger than five years was influenced by maternal education [44] and the socioeconomic status of the household [45]. These findings underpin the notion that educated mothers may have more information in implementing optimal IYCF messages [46–49]. In addition, studies from Ethiopia [50], South Africa [51] and sub-Saharan Africa [50,52] have demonstrated that high maternal education was associated with the introduction of complementary foods that may lead to better childhood nutritional status and healthy infants. Conversely, studies from Lebanon [53] and Nepal [54] suggested that the level of a mother’s education did not influence childhood stunting. The observed differences in the findings may be due to methodological differences, including the use of a limited sample size or the non-adjustment for potential cofactors [53,54].

Globally, socioeconomic variabilities have been shown to be one of the social determinants of health as it influences not only childhood nutrition but also the health of the family [34,51,55,56]. Our study indicated that children from the lowest household wealth category were more likely to be stunted compared to those from middle, richer and rich households. Similarly, studies conducted in South Africa [51], Kenya [55], Philippines [56] and sub–Saharan Africa [34] demonstrated that lower household wealth categories were associated with an increased risk of stunting compared to richer households. Household income influences the nutrition of the family and the baby, due to insufficient intake of food and decreased ability to practise safe-food handling hygiene or to appropriately store food, making it one of the major factors affecting the level of stunting in many LMICs [34,51,55,56].

In the Demographic and Health Survey project, the perceived birth size of the baby serves as a proxy for birth weight, where a mother’s perceived size of her baby plays an important role in determining the nutritional status of the child in the short- and long-term [57]. In the present study, babies perceived to be of small birth size by their mothers were more likely to be stunted compared to those perceived to be large or average, consistent with evidence from Tanzania [58], Nigeria [59], Burundi [28], Bangladesh [60], Nepal [61] and regional India [36]. These studies indicated that perceived baby size (small) was associated with childhood stunting. This finding suggests that there may be benefits in combining appropriate antenatal care services and maternal education programs regarding optimal complementary feeding practices so that babies born in India can grow up to lead healthy lives.

### Policy implications of the study findings

The present study has policy implications for researchers, health education officers, health administrators, health professionals and the public in India. The study provides nationally representative evidence on the relationship between the delayed introduction of complementary foods and associated factors with stunting and severe stunting among infants aged 6–8 months. In India, recent reports suggest that the Indian government support for essential maternal and child health (MCH) interventions is strong and increasing, demonstrable by various new MCH programs. These include the HealthPhone [62], Integrated Child Development Scheme (ICDS) [63], Prime Minister’s Overarching Scheme for Holistic Nourishment (POSHAN) Abhiyaan [64] and cash transfer schemes such as Janani Suraksha Yojna (JSY) [65]. The improvement in the nutritional status of Indian children, maternal health and parental education is at the core of these programs. Specifically, these initiatives have financial incentives and access to food supplementation from the government to mothers and their households. For example, the Government of India’s HealthPhone program focuses on a holistic approach to the health of infants, children, adolescent girls and the mothers to educate and promote optimal complementary feeding practices with a view to reducing adverse health outcomes such as stunting [62,64]. The Indian government aims to reduce stunting by 40% by the year 2025 in line with the WHO global nutrition targets 2025 to improve child health and well-being [66]. Additionally, the partnership for Water Sanitation and Hygiene (WASH) between the United States Agency for International Development and the Government of India would also have great benefit for child’s health and development in India [67].

Maternal autonomy and socioeconomic status have been associated with positive childhood nutritional outcomes in various national and sub-national studies [68–71]. Our study suggests that interventions and financial incentives that encourage the appropriate introduction of complementary foods to infants of vulnerable mothers, including those with no education and limited resources, may be helpful in reducing stunting by the year 2025 in India. Despite the current MCH interventions in India, it is unclear whether these services have substantially impacted infant nutritional outcomes in the country. Further studies that evaluate the impact of these initiatives in relation to optimal infant feeding practices may be needed to facilitate timely refinement of those programs to high-priority populations and also allow for efficient use of resources.

### Strengths and limitations of the study

The study had a number of methodological limitations which should be taken into account when interpreting the results. First, the potential effect of recall bias may have affected the study results as the survey information were self–reported by mothers through interviews. However, we restricted our analyses to the youngest living children aged 6–8 months, living with the respondent (woman aged 15–49 years) to reduce the potential impact of recall bias, consistent with previous studies [3,25,72]. Second, there could have been a misclassification in the exposures (e.g. number of ANC visits by mothers), which could result in the underestimation or overestimation of the observed associations. Third, our inability to analyse all potential cofactors (such as food insecurity, culture relating to and/or local variations of infant feeding) may have affected the association between the exposure variables and the study outcomes. Lastly, the cross-sectional nature of the data used – collected simultaneously – makes it impossible to establish a temporal association between the exposures and the outcomes.

Despite these limitations, the major strength is in the use of the most recent nationally representative data (NFHS–4). The NFHS–4 data are potentially more nationally representative of the Indian population compared to the previous NFHS due to the large sample population and methodology employed [10,39]. Since the current results are more recent and better represents the current Indian population, the evidence is potentially more beneficial to guide national policy interventions relating to stunting prevention. Also, because the data were collected by trained health personnel who used a validated questionnaire, the study findings are less likely to be influenced by measurement bias. Finally, selection bias is also unlikely to affect the study findings given the high response rates in the survey (94.0–99.6%) [10,39,57].

## Conclusion

The study indicated that delayed introduction of complementary foods was associated with stunting and severe stunting among Indian infants aged 6–8 months. Other modifiable factors associated with stunting and severe stunting included a lack of maternal education and lower household wealth. National nutrition efforts need to be context-specific in India to improve the introduction of complementary foods among infants (particularly those aged 6–8 months), while also considering the modifiable maternal vulnerabilities such as poor household wealth and no maternal schooling.

## Paper context

There is limited evidence on the broader impact of delayed introduction of complementary foods in India. This paper examined the relationship between the delayed introduction of complementary foods and stunting and severe stunting among infants aged 6–8 months in India. Our study revealed that delayed introduction of complementary foods was associated with stunting and severe stunting among infants in India and supports calls for improvements in national childhood nutrition policy actions.
